# Association between *Cyclooxygenase-2* and* Indoleamine 2,3-Dioxygenase* Expression in Breast Cancer Patients from Pakistan

**DOI:** 10.31557/APJCP.2019.20.11.3521

**Published:** 2019

**Authors:** Kashif Asghar, Asif Loya, Iftikhar Ali Rana, Muhammad Abu Bakar, Asim Farooq, Muhammad Tahseen, Muhammad Ishaq, Muhammad Usman Rashid

**Affiliations:** 1 *Department of Basic Sciences, *; 2 *Department of Pathology, *; 3 *Department of Cancer Registry and Clinical Data Management, *; 4 *Department of Clinical Research, Shaukat Khanum Memorial Cancer Hospital and Research Centre (SKMCH and RC), Lahore, Pakistan. *

**Keywords:** Cyclooxygenase-2, indoleamine 2- 3-dioxygenase, breast cancer, Pakistan

## Abstract

**Background:**

Tumors use several immunosuppressive mechanisms to evade immune destruction. *Cyclooxygenase-2 (COX-2)* expression may be a driver of immunosuppression in breast cancer, but the mechanisms involved remain elusive. *COX-2* expression induces the expression of *indoleamine 2,3 dioxygenase (IDO)* in tumor cells. *IDO* is an immunosuppressive enzyme which is involved in tumor immune escape mechanisms in breast cancer. Our aim was to evaluate the association between *COX-2* and* IDO* expression to find evidence of immunosuppression in Pakistani breast cancer patients.

**Methods:**

Immunohistochemical analysis was performed to evaluate the expression of *COX-2, IDO, *estrogen receptor (ER), progesterone receptor (PR) and human epidermal growth factor receptor 2 (HER2) on formalin-fixed paraffin-embedded breast cancer tissues of 100 patients. Univariable and multivariable logistic regression model was used to identify the independent risk factors of* COX-2.*

**Results:**

A total of 100 patients were included with a mean age and standard deviation of 48.28 ± 11.83. A significant association was observed among *COX-2*, *IDO*, ER, PR and tumor grade. In multivariable analysis, three variables were identified as significant independent risk factors for high *COX-2: IDO* expression high; [adjusted odds ratio (AOR) 6.51; 95% confidence interval (CI) (2.00-21.20), p=0.001], ER; [AOR 5.62; 95% CI (1.80-17.84), p=0.002] and age [AOR 1.04; 95% CI (1.00-1.10), p=0.05] respectively.

**Conclusion:**

Our data showed that high *IDO* expression is associated with high *COX-2* expression in Pakistani breast cancer patients. The co-expression of both enzymes may suggest their role in disease pathogenesis. Hence the concurrent targeting of *COX-2* and *IDO* may be a promising therapy for breast cancer.

## Introduction

The cyclooxygenase (COX) family of enzymes contains two members (*COX-1 *and *COX-2*) (Chen et al., 2014). *COX-1* is expressed ubiquitously (Williams and DuBois, 1996). *COX-2* is expressed in distinct tissues and is involved in inflammatory processes (Herschman, 1996). Chronic inflammation is well known to be linked with cancer progression (Howe, 2007). Recent emerging epidemiologic, preclinical, and clinical data suggest that *COX-2* up-regulation is a fundamental step in carcinogenesis (Cao and Prescott, 2002) and tumor angiogenesis (Davies et al., 2003). Elevated *COX-2 *expression has been detected in human breast tumor tissues (Ristimaki et al., 2002). 

The age-associated increase in *COX-2* activity has been noticed in animal models (Claycombe et al., 2002). Elevated level of COX-derived products has been identified in platelets and peripheral blood mononuclear cells (PBMCs) in elderly humans (Vericel et al., 1988; Meydani et al., 1990). Furthermore,* COX-2* up regulation is associated with distinct pathological features such as large tumor size, high tumor grade and metastasis (Ristimaki et al., 2002; Wulfing et al., 2003). *COX-2 *induces aromatase in breast tissue (Salhab et al., 2007). Aromatase activity increases estrogen levels (Vienonen et al., 2002). Consequently, *COX-2* expression increases the estrogen levels and subsequently tumor progression in hormone receptor positive breast cancer (Hoellen et al., 2011).

Tryptophan catabolism is linked with immunosuppression in the tumor microenvironment. *Indoleamine 2, 3-dioxygenase (IDO) *is a heme containing tryptophan-degrading enzyme (McGaha et al., 2012). *IDO* overexpression in tumors results in prompt conversion of tryptophan into kynurenine. Tryptophan depletion and enhanced levels of kynurenine play pivotal role in immunosuppression (McGaha et al., 2012; van Baren and Van den Eynde, 2015).* IDO* expression is involved in breast tumor growth and pulmonary metastasis (Levina et al., 2012). Furthermore, high *IDO* expression is significantly linked with overall decreased patient survival (Asghar et al., 2019). *COX-2* and *IDO* promote breast cancer progression (Chen et al., 2014). The current study has been conducted in Pakistan, a country with one of the high incidence of breast cancer in its region, to investigate the association between *COX-2* and *IDO* expression in breast cancer patients. 

## Materials and Methods

*Tumor tissue specimens*

A retrospective study was performed on formalin-fixed paraffin-embedded (FFPE) tumor specimens of breast cancer patients (n=100). These specimens were retrieved from pathology department, at Shaukat Khanum Memorial Cancer Hospital and Research Centre (SKMCH and RC) Lahore, Pakistan. The patients selected for the current study were diagnosed with breast cancer between 2007 and 2009. All the patients were treatment naïve. Tumor grade was allocated using the Nottingham Histologic Score. IHC analysis of estrogen receptor (ER), progesterone receptor (PR) and human epidermal growth factor receptor 2 (HER2) expressions were conducted and interpreted using standard methods (Chen et al., 2010). The comprehensive information about the clinico-pathological characteristics was retrieved from medical records and pathology reports. 

*Ethical approval for retrospective study*

The current study (#IRB-16-08) was approved by the Institutional Review Board (IRB) of the SKMCH and RC. This study was exempted for informed consent by IRB (SKMCH and RC) which is in compliance with the Declaration of Helsinki. 

*Immunohistochemical staining*

Slides were stained using Bond III Leica automated system (Leica Biosystems Melbourne, Australia) as per manufacturer’s protocol. Two sections of FFPE tumor specimens of the same patients were attained, and slides were deparaffinized on the automated system with Bond Dewax solution (Leica Biosystems). Briefly, heat induced epitope retrieval was performed with Bond ER -2 (Leica Biosystems), for 20 min. Both the primary antibodies *COX-2* (abcam, # ab15191, anti-*COX-2* antibody) and *IDO1 *(abcam, # ab55305, anti-Indoleamine 2,3-dioxygenase antibody) were used at a 1:200 concentration. Tissue sections were incubated for 5 min with appropriate primary antibody in diluent Bond (Leica Biosystems). Antibody labeling was visualized by BondTM polymer refine detection kit. Then incubated with post primary rabbit anti mouse IgG for 8 min and subsequently, incubated with polymer anti-rabbit poly-HRP-IgG for 8 min. DAB 3, 3’-diaminobenzidine tetrahydrochloride hydrate was used as chromogen. Slides were dehydrated and cover slipped as per our earlier described laboratory protocol (Asghar et al., 2019). Slides were visualized by an optical microscope (Provis AX-70, Olympus, Melville, NY).

*Evaluation of COX-2 and IDO scoring*

It was a blind histopathologic assessment. The slides were evaluated by pathologists. The *COX-2* and *IDO* immunostaining were examined by using the H-score, well-defined by the following equation: H-score = ΣPi (i + 1), as described previously (Chan et al., 2012), Pi is the percentage of stained tumor cells and i is the intensity of cytoplasmic staining of tumor cells (0 to 3+). 

*Statistical analysis*

Statistical analysis was carried out using SPSS software (version 20.0; SPSS, Chicago, IL, USA). Percentages (proportions) were used for categorical variables while mean and standard deviation were used for continuous variables. Bivariate analysis was done using chi-square or fisher exact test (when necessary). For continuous explanatory variables such as age, independent t-test was performed. Univariable and multivariable logistic regression model was used to identify the risk factors.

**Figure 1:**
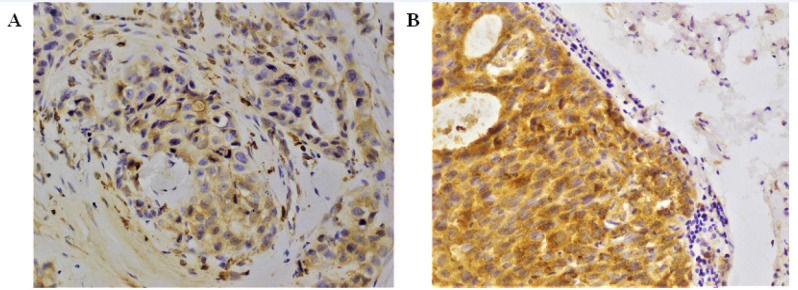
*COX-2* Immunostaining Images. *COX-2* expression in breast cancer patients (n=100) tissues was assessed by immunohistochemistry (A) Low *COX-2* expression was observed in ductal carcinoma. (B) High *COX-2* expression was detected in invasive ductal carcinoma. The staining was cytoplasmic. Images were captured at x 40 magnification

**Table 1:** Demographic Characteristics versus Low and High *COX-2* Scores

Variables	Characteristics	Total 100 (100.0%)	Low *COX-2* 41 (41.0%)	High *COX-2* 59 (59.0%)	p-value
Age	Mean ± SD	48.28 ± 11.83	45.02 ± 11.20	50.54 ± 11.82	0.02*
Region	Punjab	88 (88.0)	35 (39.8)	53 (60.2)	0.5
	Khyber Pakhtunkhwa	7.0 (7.0)	3 (42.9)	4 (57.1)	
	Kashmir	3.0 (3.0)	1 (33.3)	2 (66.7)	
	Sindh	2.0 (2.0)	2 (100.0)	0 (0.0)	

values indicate statistical significance p< 0.05; SD, Standard deviation

**Table 2:** Clinicopathological Characteristics versus Low and High *COX-2 *Scores

Variables	Characteristics	Total 100 (100.0%)	Low *COX-2* 41 (41.0%)	High *COX-2* 59 (59.0%)	p-value
IDO Score	Low	24 (24.0)	16 (66.7)	8 (33.3)	0.004*
	High	76 (76.0)	25 (32.9)	51 (67.1)	
	Total	100 (100.0)	41 (41.0)	59 (59.0)	
Estrogen receptor	Negative	69 (69.0)	35 (50.7)	34 (49.3)	0.003*
	Positive	31 (31.0)	6 (19.4)	25 (80.6)	
	Total	100 (100.0)	41 (41.0)	59 (59.0)	
Progesterone receptor	Negative	74 (74.0)	37 (50.0)	37 (50.0)	0.002*
	Positive	26 (26.0)	4 (15.4)	22 (84.6)	
	Total	100 (100.0)	41 (41.0)	59 (59.0)	
HER2–neu receptor	Negative	74 (74.0)	33 (44.6)	41 (55.4)	0.22
	Positive	26 (26.0)	8 (30.8)	18 (69.2)	
	Total	100 (100.0)	41 (41.0)	59 (59.0)	
Metastasis	Negative	38 (38.0)	19 (50.0)	19 (50.0)	0.21
	Positive	49 (49.0)	18 (36.7)	31 (63.3)	
	Total	87 (87.0)	37 (42.5)	50 (57.5)	
Grade	II	36 (36.0)	10 (27.8)	26 (72.2)	0.02*
	III	56 (56.0)	29 (51.8)	27 (48.2)	
	Total	92 (92.0)	39 (42.4)	53 (57.6)	
Nodes	N0	37 (37.0)	18 (48.6)	19 (51.4)	0.17
	N1	24 (24.0)	7 (29.2)	17 (70.8)	
	N2	13 (13.0)	4 (30.8)	9 (69.2)	
	N3	13 (13.0)	8 (61.5)	5 (38.5)	
	Total	87 (87.0)	37 (42.5)	50 (57.5)	
Histology	Ductal	91 (91.0)	38 (41.8)	53 (58.2)	0.82
	Mammary	6 (6.0)	2 (33.3)	4 (66.7)	
	Lobular	2 (2.0)	1 (50.0)	1 (50.0)	
	Metaplastic	1 (1.0)	0 (0.0)	1 (100.0)	
	Total	100 (100.0)	41 (41.0)	59 (59.0)	
Tumor Size	T1	7 (7.0)	2 (28.6)	5 (71.4)	0.34
	T2	49 (49.0)	18 (36.7)	31 (63.3)	
	T3	8 (8.0)	5 (62.5)	3 (37.5)	
	Total	64 (64.0)	25 (39.1)	39 (60.9)	

*values indicate statistical significance p< 0.05; IDO, Indoleamine 2,3-dioxygenase

**Figure 2:**
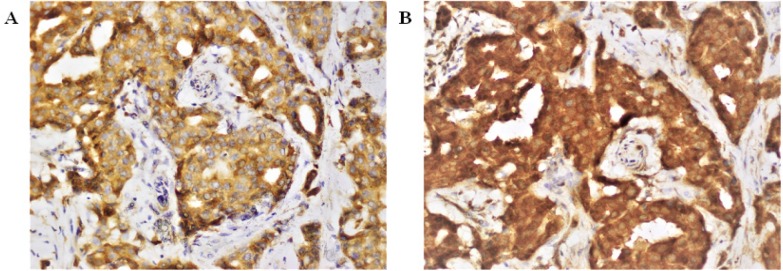
*COX-2* and *IDO* co-Expression. (A) Strong *COX-2* expression was detected in invasive ductal carcinoma. Sections from the same breast cancer patients were stained for *IDO* (B) Strong and diffuse *IDO* staining in invasive ductal tumor cells. Both staining were cytoplasmic. Images were captured at x 40 magnification

**Table 3:** Risk Factors of *COX-2 *High Expression

Variables	Characteristics	Univariable analysis odds ratio (95% CI), p-value	Multivariable analysis odds ratio (95% CI), p-value
Age (years)	Mean ± SD	1.04 (1.00-1.10), 0.02	1.04 (1.00-1.10), 0.05*
Estrogen receptor	Negative	Ref	Ref
	Positive	4.30 (1.60-11.76), 0.01	5.62 (1.80-17.84), 0.002*
*IDO* Score	Low	Ref	Ref
	High	4.10 (1.54-10.81), 0.01	6.51 (2.00-21.20), 0.001*

*values indicate statistical significance p< 0.05; IDO, Indoleamine 2,3-dioxygenase

## Results

*Demographic characteristics versus low and high COX-2 scores*

Demographic characteristics of 100 breast cancer patients are summarized in Table 1. Overall the mean age at breast cancer diagnosis was 48.28 ± 11.83, and there was a mean difference of age in low and high* COX-2 *expression (p=0.02). Majority of patients belonged to the Punjab region (88%). 

*Clinicopathological characteristics versus low and high COX-2 scores*

In order to examine the association between *COX-2* and IDO, we categorized the patients into *COX-2* low versus high. There was a statistically significant association of *COX-2* with *IDO* expression (p = 0.004), ER (p = 0.003), PR (p = 0.002) and tumor grade (p= 0.02) respectively (Table 2).

*COX-2 and IDO immunostaining*

To investigate the expression of *COX-2* and *IDO*, FFPE tumor specimens (n=100) of same patients were selected. Out of 100 tumor specimen, *COX-2* high, and low scores were 59%, and 41%, respectively (Figure 1). *IDO* positivity was observed in all breast tumor specimens. *COX-2* and *IDO* co-expression is shown in Figure 2. 

*Risk factors of COX-2 high expression*

Table 3 summarizes various clinical and pathological factors that were included in the univariable and multivariable analyses to identify the *COX-2* association with *IDO* expression. In multivariable analysis, three variables were identified as significant independent risk factors for high *COX-2: IDO* expression high; [adjusted odds ratio (AOR) 6.51; 95% confidence interval (CI) (2.00- 21.20), p=0.001], ER; [AOR 5.62; 95% CI (1.80- 17.84), p=0.002] and age [AOR 1.04; 95% CI (1.00- 1.10), 0.05. 

## Discussion

Previous studies established that *IDO* overexpression is involved in tumor immune escape in various cancers (Muller et al., 2005; Mansfield et al., 2009). It has been documented that *IDO* and *COX-2* promote breast cancer progression (Chen et al., 2014). To the best of our knowledge, this is first study which revealed that high *IDO *expression is associated with high *COX-2* expression in Pakistani breast cancer patients; coherent with the findings demonstrated by Mei et al., (2012). It is well established that *COX-2* expression induces constitutive expression of *IDO* in human tumor cells (Hennequart et al., 2017). But interestingly Mei et al., (2012) demonstrated that IDO inhibitors suppressed the *COX-2* expression and *IDO* may be involved in endometriosis pathogenesis via promoting *COX-2*. Our data showed as well that high* IDO* expression is associated with *COX-2* expression which might play a role in breast cancer pathogenesis. 

*COX-2* upregulation is involved in age-related dysregulation of the immune responses (Wu and Meydani, 2004). Siironen et al., (2004) demonstrated that an increase in the *COX-2* expression is associated with age in papillary thyroid cancer patients. Our data revealed that increase in the *COX-2* expression is associated with age in Pakistani breast cancer patients. In addition, it has been established that ER-positive breast cancer may evolve from low to high grade (Lopez-Garcia et al., 2010; Natrajan et al., 2010). *COX-2* expression upregulates transcription of aromatase and consequently stimulates tumor cell progression in ER-positive breast cancer (Diaz-Cruz et al., 2005). Our results are in agreement that high *COX-2* expression is associated with ER-positive breast cancer. 

As we aimed to investigate the association between *COX-2* and *IDO* expression in breast cancer patients from Pakistan, a country with high incidence of breast cancer cases, our data showed the high *IDO* expression is associated with high *COX-2* expression. The co-expression of both enzymes may suggest their role in disease pathogenesis. Basu et al., (2004) suggested that *COX-2*-mediated immunosuppression through IDO regulation may act as therapeutic target for the development of future cancer vaccines. Chen et al., (2014) demonstrated that stromal *IDO *upregulation is associated with overexpression of *COX-2*. Previous studies established that increase in* COX-2* expression lead to increase in *IDO* expression. Our data is demonstrating that an increase in *IDO* expression is associated with an increase in *COX-2* expression. A significant association is present between *IDO* and *COX-2* expression in Pakistani breast cancer patients. The data presented in this study will certainly serve as a useful addition to the already available knowledge, while the molecular mechanisms underlying *IDO* mediated *COX-2* expression need further investigation. Hence the simultaneous targeting of *IDO *and *COX-2* may be a promising therapy for breast cancer.
